# Rewiring T Cell Metabolism to Enhance CAR T Cell Function in Solid Tumor Microenvironments

**DOI:** 10.3390/pharmaceutics17121520

**Published:** 2025-11-26

**Authors:** Alex Wade Song, Xiaotong Song

**Affiliations:** 1 Cellula BioPharma, Inc., 2450 Holcombe Blvd Suite J, Houston, TX 77021, USA; info@cellula-biopharma.com; 2 Department of Translational Medical Sciences, College of Medicine, Texas A&M University, 2121 W Holcombe Blvd, Houston, TX 77030, USA; 3 Center for Infectious and Inflammatory Diseases, Institute of Bioscience and Technology, Texas A&M University, 2121 W Holcombe Blvd, Houston, TX 77030, USA

**Keywords:** CAR T cells, tumor microenvironment, metabolic reprogramming, hypoxia, adenosine

## Abstract

**Background/Objectives**: Chimeric antigen receptor (CAR) T cells have shown remarkable clinical success in certain blood cancers but remain largely ineffective in solid tumors. A major reason for this limitation is the hostile tumor microenvironment, which restricts oxygen and nutrients while producing toxic metabolites that suppress immune cell activity. This review aims to examine how targeted metabolic reprogramming can overcome these barriers and improve CAR T cell performance. **Methods**: We evaluated preclinical and translational studies that focused on engineering CAR T cells to resist hypoxia, improve nutrient utilization, reduce metabolic exhaustion, and counteract suppressive metabolites in solid tumors. **Results**: Emerging strategies include engineering resistance to low oxygen and high lactate, enhancing nutrient uptake through transporter overexpression, and blocking inhibitory pathways such as those driven by adenosine. These approaches improve CAR T cell persistence, memory formation, and cytotoxic function in challenging tumor environments. **Conclusions:** Integrating metabolic reprogramming with conventional CAR design is essential to unlock the full potential of CAR T therapy against solid tumors. Continued innovation in this area will be critical for translating laboratory advances into effective clinical treatments.

## 1. Introduction

The advent of CAR T cell therapy has marked a significant advancement in oncology, achieving remarkable success in the treatment of refractory B cell hematologic malignancies [1,2,3,4,5,6,7]. Landmark clinical trials have demonstrated unprecedented response rates in patients with acute lymphoblastic leukemia and diffuse large B cell lymphoma, leading to the rapid U.S. Food and Drug Administration (FDA) approval of several CAR T products. This breakthrough validates the potent capacity of genetically engineered T cells to eradicate cancer.

However, the translation of this spectacular success to solid tumors has been markedly limited [8,9,10]. Despite shared principles of target antigen recognition and cytotoxicity, CAR T cells encounter numerous challenges within the solid tumor microenvironment (TME) that are largely absent in the circulation. These challenges include physical barriers like dense fibrotic stroma that impedes infiltration, antigenic heterogeneity leading to immune escape, and most critically, a highly immunosuppressive and metabolically unfavorable tumor microenvironment.

This immunosuppressive TME represents an area of intense metabolic competition between tumor cells and immune cells designed to cripple effector immune cells [11,12,13,14]. Tumor cells, characterized by their hyperactive and dysregulated metabolism (the Warburg effect), aggressively consume essential nutrients like glucose and glutamine, creating a state of nutrient deprivation [15,16,17,18,19,20]. Concurrently, they extrude waste products, including lactic acid, resulting in extracellular acidosis [21,22,23]. Furthermore, the TME is often profoundly hypoxic and enriched with potent immunosuppressive metabolites such as adenosine and kynurenines, generated by tumor and stromal cells [24,25,26,27,28,29,30,31,32]. This metabolic landscape is not a passive backdrop but an active mechanism of immune evasion. It directly sabotages CAR T cell function by impairing cytokine production, cytotoxic degranulation, and proliferative capacity, ultimately driving T cells toward a state of functional exhaustion and apoptosis [13,33,34].

Consequently, the failure of CAR T cells in solid tumors is not merely an engineering problem of antigen recognition, but a fundamental problem of cellular fuel and survival. It is increasingly clear that for CAR T cells to become a viable therapeutic option for solid malignancies, we must expand our focus beyond optimizing the synthetic CAR receptor itself. Future advancements are expected through the modification of intrinsic CAR T cell biology to withstand this hostile metabolic siege.

This review was conducted as a narrative synthesis with the aim of providing an up-to-date overview of CAR T cell metabolism in the context of T cell subsets and CAR design. We performed literature searches in PubMed, Web of Science, and Scopus using combinations of the following keywords: “CAR T cell”, “chimeric antigen receptor”, “T cell metabolism”, “glycolysis”, “oxidative phosphorylation”, “costimulatory domain”, “Cluster of Differentiation 28 (CD28)”, “4-1BB”, “tonic signaling”, “single-cell RNA-sequencing”, and “bioinformatics”. We considered publications from January 1970 through to October 2025 to ensure coverage of seminal and recent advances in the field. Studies were included if they provided mechanistic insights into T cell or CAR T cell metabolism, clinical or translational relevance, or detailed bioinformatic analyses. Preference was given to original research articles and high-impact reviews. Studies were excluded if they lacked relevance to T cell metabolism, focused solely on other cell types, or were published only as abstracts or non-peer-reviewed sources. In this review, we first delineate the metabolic complexities of the solid TME and its detrimental impact on T cell physiology. We then outline the core principles of T cell immunometabolism, contrasting the metabolic states of activation, memory, and exhaustion. The central focus is a comprehensive analysis of innovative strategies to reprogram CAR T cell metabolism—through genetic, pharmacological, and manufacturing interventions—to enhance their persistence, stemness, and effector function. Finally, we discuss the translational challenges, safety considerations, future directions of this promising approach, arguing that endowing CAR T cells with metabolic fitness is not merely an additive enhancement but an essential requirement for conquering solid tumors.

## 2. The Metabolic Landscape of the Solid Tumor Microenvironment

The solid TME is not a passive bystander but a dynamically evolved fortress, meticulously engineered by cancer cells to foster their own proliferation while actively dismantling anti-tumor immunity. For adoptively transferred CAR T cells, infiltrating this niche is akin to a soldier entering a battlefield rigged with traps, deprived of supplies, and poisoned with toxins. This metabolic sabotage constitutes one of the most formidable and non-redundant barriers to efficacy, and its features are characterized by a profound depletion of essential nutrients and a concurrent accumulation of toxic by-products (Table 1).

### 2.1. Nutrient Depletion: Starving the Invaders

The most starkly evident feature is nutrient deprivation. Tumor cells, exhibiting a voracious and inefficient appetite due to the Warburg effect, consume glucose at an alarming rate, creating a state of intense competition and profound hypoglycemia [17,35]. This is catastrophic for infiltrating T cells, whose activation, proliferation, and effector functions are critically dependent on high glycolytic flux. Glucose starvation impairs glycolytic capacity, leading to a direct reduction in interferon-gamma (IFN-γ) production, diminished cytotoxic degranulation, and ultimately, functional paralysis. Beyond glucose, the TME is also scavenged of critical amino acids. The enzyme indoleamine 2,3-dioxygenase 1 (IDO1), highly expressed by tumor cells and immunosuppressive myeloid cells like dendritic cells and macrophages, catabolizes the essential amino acid tryptophan into kynurenine [36,37]. This creates a dual immunosuppressive mechanism: tryptophan depletion itself stalls T cell proliferation by activating the integrated stress response kinase, General Control Nonderepressible 2 kinase (GCN2), while the resulting kynurenines activate the aryl hydrocarbon receptor (AhR) in T cells, promoting regulatory T cell (Treg) differentiation, inducing Fas-mediated apoptosis, and directly driving exhaustion. Similarly, the amino acid arginine is depleted by the overexpression of arginase 1 (ARG1) in myeloid-derived suppressor cells (MDSCs) and some tumor cells [38,39,40,41]. Arginine starvation in T cells leads to the downregulation of the CD3ζ chain, cell cycle arrest in the G0-G1 phase, and a profound suppression of antigen-specific T cell responses.

### 2.2. Accumulation of Suppressive Metabolites: Impeding Immune Cell Infiltration and Activity Through Suppressive Mechanisms

Simultaneously, the TME is poisoned by the accumulation of immunosuppressive waste products. The high glycolytic rate of tumor cells leads to an enormous export of lactic acid, acidifying the extracellular pH to levels as low as 6.0–6.5 [21,22,23]. This extracellular acidosis is not merely an incidental by-product but an active suppressor of immunity. It inhibits mTOR (mechanistic Target of Rapamycin; mTOR integrates signals from nutrients, growth factors, and cellular energy status to regulate T cell growth, proliferation, and differentiation, particularly driving anabolic metabolism for effector functions) activity and glycolytic enzyme function, directly impairing the central metabolic pathway of effector T cells [42,43,44]. It also suppresses nuclear factor of activated T cells (NFAT) signaling, leading to reduced cytokine production and cytotoxicity, and inhibits T cell motility and migration, trapping them in a dysfunctional state [45].

In parallel, the hypoxic core of the TME fuels the generation of extracellular adenosine (ADO), a potent immunosuppressive metabolite [46,47,48,49,50]. The ectoenzymes CD39 (which hydrolyzes Adenosine Triphosphate (ATP)/Adenosine Diphosphate (ADP) to Adenosine Monophosphate (AMP)) and CD73 (which dephosphorylates AMP to ADO) are highly expressed on the surface of tumor cells, cancer-associated fibroblasts (CAFs), and regulatory immune cells [48]. Signaling through the A2A adenosine receptor (A2AR) on T cells activates a potent immunosuppressive cascade involving cyclic AMP (cAMP) and protein kinase A (PKA) [51,52,53]. This signaling axis inhibits T cell receptor (TCR) activity, blocks cytokine production, reduces proliferation, and impairs cytotoxicity, while simultaneously promoting the expression of immune checkpoint molecules such as Programmed Cell Death Protein-1 (PD-1) and Cytotoxic T-Lymphocyte-Associated Protein 4 (CTLA-4). In this way, ADO acts as both a brake on effector function and a driver of exhaustion.

Compounding the problem, adenosine accumulation forms a self-reinforcing loop: immune activation releases extracellular ATP, which is sequentially degraded by CD39 and CD73 into ADO, thereby amplifying local immunosuppression. This creates a metabolically “poisoned well”, where every attempt at immune activation paradoxically strengthens inhibitory signaling.

Importantly, recent studies suggest that ADO is not purely suppressive. Through adenosine deaminase (ADA)-mediated conversion, extracellular adenosine can be degraded into inosine (Ino), a metabolite that is not immunosuppressive but rather can serve as an alternative fuel source for T cells [54,55]. Ino enters central carbon metabolism through purine salvage pathways and can support both glycolysis and oxidative phosphorylation under glucose-limiting conditions [56]. Thus, while ADO accumulation in the TME undermines immunity through A2AR signaling, its enzymatic conversion to inosine provides an exploitable metabolic vulnerability—one that can be leveraged by engineered CAR T cells to both detoxify the TME and harness inosine as an auxiliary energy substrate.

### 2.3. Hypoxia: Suffocating the Response

Finally, the dysregulated growth of solid tumors outstrips its vascular supply, creating vast hypoxic regions. Hypoxia stabilizes hypoxia-inducible factors (HIFs), which drive a pro-tumorigenic program [57,58]. HIF-1α is stabilized under hypoxic conditions and shifts T cells toward glycolytic metabolism, facilitating effector differentiation while linking immune activation to oxygen availability in the microenvironment. In tumor cells, HIF-1α promotes angiogenesis, invasion, metastasis, and further enhances glycolytic metabolism, exacerbating the problems of nutrient depletion and acidosis. In T cells, hypoxia and HIF-1α accumulation directly impair function by driving cells toward a terminally exhausted state characterized by the high and coordinated expression of multiple inhibitory receptors, e.g., PD-1, Lymphocyte-Activation Gene 3 (LAG-3), and T cell immunoglobulin and mucin-domain containing-3 (TIM-3). Moreover, hypoxia suppresses mitochondrial respiration and oxidative metabolism, the preferred energy pathway for developing long-lived memory T cells, thereby favoring the generation of short-lived effector cells that rapidly undergo activation-induced cell death [44,59,60,61,62]. The resulting metabolic landscape exhibits highly efficient immune evasion strategies, simultaneously starving T cells of essential fuels, poisoning them with waste products, and suffocating their capacity for long-term function and memory.

**Table 1:** Key Metabolic Features of the Immunosuppressive TME.

Metabolic Feature	Primary Mechanism	Direct Impact on CAR T Cells
Hypoglycemia [15,17]	Warburg effect in tumor cells	Impairs glycolysis, cytokine production, and cytotoxicity
Acidosis [42,49]	Export of lactic acid	Suppresses mTOR signaling, motility, and effector function
Tryptophan Depletion [36,37]	IDO1/Kynurenine pathway	Arrests proliferation; induces apoptosis and Treg differentiation
Arginine Depletion [38,40]	ARG1 activity	Downregulates CD3ζ chain; causes cell cycle arrest
Adenosine Rich [47]	CD39/CD73 ectoenzyme activity	Engages A2AR, suppressing TCR signaling and promoting exhaustion
Hypoxia [49]	Dysregulated tumor growth	Stabilizes HIF-1α, driving exhaustion and suppressing memory formation
High ROS [59,63]	Dysfunctional tumor metabolism	Causes oxidative damage, inhibiting function and inducing apoptosis

The hostile metabolic landscape of solid tumors imposes multiple barriers on CAR T cell function. Nutrient deprivation, hypoxia, and the accumulation of metabolic by-products (such as adenosine and lactate) impair CAR T cell trafficking and infiltration by reducing chemokine responsiveness and cytoskeletal dynamics. Within the tumor, limited access to glucose and amino acids curtails effector metabolic programs necessary for sustained proliferation and cytotoxic activity, while hypoxia diminishes mitochondrial function, leading to reduced survival and persistence. High metabolic stress also promotes the rapid acquisition of a dysfunctional or “exhausted” phenotype, characterized by impaired cytokine production and diminished killing capacity. Furthermore, chronic antigen exposure in a metabolically suppressive milieu accelerates T cell fatigue and loss of efficacy. These factors collectively restrict CAR T cell performance in solid tumors, and overcoming these metabolic limitations remains a critical area for therapeutic innovation.

## 3. Principles of T Cell Immunometabolism

To rationally design CAR T cells capable of withstanding the metabolic siege of the tumor microenvironment, a deep understanding of the fundamental metabolic programs that govern T cell fate and function is essential. T cell activation, differentiation, and memory formation are processes inextricably linked to dynamic and precisely regulated shifts in cellular metabolism. These shifts are not merely secondary consequences of signaling events but are primary drivers of T cell phenotype and function. The immunosuppressive TME directly hijacks and disrupts these critical metabolic processes, leading to the dysfunctional state observed in tumor-infiltrating lymphocytes.

### 3.1. The Metabolic Journey of a T Cell

The metabolic journey of a T cell begins in a quiescent, naïve state (Table 2). Naïve T cells, tasked with long-term immune surveillance, are metabolically quiet and catabolic. Their minimal energy demands are met through efficient oxidative phosphorylation (OXPHOS) within the mitochondria, fueled by a mix of fatty acid oxidation (FAO) and pyruvate generated from low levels of glycolysis [62,64,65,66,67,68,69,70,71,72]. This state of metabolic efficiency, supported by AMP-activated protein kinase (AMPK) signaling, allows them to maintain readiness for years while circulating through the body [11,73]. As a cellular energy sensor, AMPK promotes catabolic metabolism and mitochondrial biogenesis, supporting T cell quiescence, survival, and the development of memory T cells under low-energy conditions. This catabolic homeostasis is shattered upon TCR engagement by cognate antigen presented on major histocompatibility complex (MHC) molecules, coupled with crucial co-stimulatory signals (e.g., CD28). This activation signal triggers a profound anabolic switch, a metabolic reprogramming as dramatic as the genetic one. To support the immense biosynthetic demands of rapid clonal expansion—the synthesis of proteins, lipids, and nucleic acids for daughter cells—and the production of effector molecules like IFN-γ, granzymes, and perforin, the cell shifts to a state of high-rate aerobic glycolysis. This process, known as the Warburg effect, involves the rapid fermentation of glucose to lactate, even in the presence of ample oxygen to support mitochondrial respiration. While highly inefficient in terms of ATP yield per glucose molecule, glycolysis provides rapid ATP generation, and most importantly, critical metabolic intermediates that serve as building blocks for macromolecular synthesis. This glycolytic burst is orchestrated by the PI3K–AKT–mTOR signaling axis. The PI3K/Akt signaling pathway promotes T cell activation and survival by stimulating glycolysis and cellular growth, and is a key mediator connecting extracellular signals to metabolic changes during immune responses. This metabolic program is the hallmark of short-lived, potent effector T cells (Teff).

Following antigen clearance, a small subset of effector cells escapes death and transitions into long-lived memory T cells (Tm). This critical differentiation step is accompanied by a metabolic shift back toward a catabolic, mitochondrial-dependent state [11]. Memory T cells downregulate glycolytic activity and enhance mitochondrial biogenesis, spare respiratory capacity (SRC), and reliance on FAO and OXPHOS. This metabolic reprogramming is not a reversion to the naïve state but an acquisition of a unique, metabolically flexible phenotype. The increased mitochondrial mass and SRC provide a readily available energy reservoir, allowing memory T cells to rapidly re-engage aerobic glycolysis and mount a powerful recall response upon secondary antigen encounter. The metabolic fitness of the mitochondrial pool, therefore, is a key determinant of a T cell’s fate, dictating whether it becomes a persistent, functional guardian or a transient, disposable effector.

### 3.2. The Metabolism of Exhaustion

In stark contrast to the robust metabolism of effective immunity lies the dysfunctional metabolism of exhaustion [12,74,75,76]. T cell exhaustion is a state of progressive and hierarchical loss of effector function, defined by the sustained expression of multiple inhibitory receptors (e.g., PD-1, TIM-3, LAG-3), reduced cytokine production, and impaired cytolytic activity. It is now unequivocally clear that exhaustion is not solely a transcriptional and epigenetic program but is also a distinct metabolic state of failure. When T cells encounter chronic antigen and inflammatory signals within the TME—a scenario perfectly mimicked by a solid tumor—and simultaneously face the metabolic stressors described in Section 2, their metabolic programs become severely crippled. Exhausted T cells exhibit significant mitochondrial dysfunction, including reduced mitochondrial mass, loss of membrane potential, fragmented morphology, and low spare respiratory capacity (SRC). They are bioenergetically bankrupt, unable to efficiently produce energy via OXPHOS even if nutrients are available. Concurrently, persistent signaling through inhibitory receptors like PD-1 suppresses PI3K–AKT–mTOR activity, blunting the anabolic glycolytic switch necessary for effector function. Caught in a state where they cannot engage glycolysis effectively nor perform OXPHOS efficiently, exhausted T cells become metabolically inert, unable to support the energy-intensive processes of killing tumor cells and proliferating. This metabolic insufficiency is both a cause and a consequence of the exhausted state, creating a vicious cycle that locks T cells into dysfunction.

An often-overlooked contributor to this metabolic collapse is the accumulation of extracellular ADO within the TME [34,47]. ADO is generated through the sequential hydrolysis of extracellular ATP by the ectonucleotidases CD39 and CD73, processes that are strongly upregulated in hypoxic and inflamed tumor beds. Elevated ADO imposes a dual barrier to T cell function: (i) as a potent immunosuppressive signal, it binds the A2A receptor on T cells, inhibiting cAMP-sensitive pathways and dampening PI3K–AKT–mTOR activity, thereby preventing glycolytic engagement; and (ii) as a metabolically unusable “dead-end” nucleoside, it displaces glucose and inosine without contributing to central carbon metabolism. Thus, high levels of extracellular adenosine simultaneously signal suppression and deny T cells access to usable nutrients.

Targeting this adenosine axis has therefore emerged as a compelling metabolic reprogramming strategy [34,56,77]. A2A receptor knockout renders T cells insensitive to adenosine-mediated suppression, though with the risk of hyperactivation. CD73 blockade prevents adenosine production altogether but does not supply alternative fuel. A more integrative approach leverages ADA1, an enzyme that irreversibly converts adenosine to inosine [78,79]. This strategy has twofold benefits: (i) it relieves immunosuppressive A2A receptor signaling by depleting extracellular adenosine, and (ii) it generates inosine, a nucleoside that can be salvaged via purine nucleoside phosphorylase into ribose-1-phosphate, entering glycolysis and the pentose phosphate pathway to support both energy production and biosynthesis under glucose deprivation. Exogenous inosine supplementation has similarly been shown to rescue T cell proliferation and effector function in glucose-limited environments, underscoring its role as a metabolic lifeline.

By reframing adenosine from an immunosuppressive “toxin” into a metabolic substrate through ADA1 activity, this axis couples immune checkpoint relief with metabolic rescue. Importantly, this mechanism enhances metabolic plasticity, endowing T cells with the capacity to adapt to fluctuating nutrient conditions within the TME. The adenosine–inosine pathway thus represents a promising frontier in the metabolic engineering of CAR T cells, directly targeting both the inhibitory signaling and the metabolic starvation that drive exhaustion.

**Table 2:** Metabolic States of T Cell Differentiation.

T Cell Subset	Primary Metabolic Pathway(s)	Metabolic Characteristics	Functional Outcome
Naïve T Cell [80]	OXPHOS, FAO	Catabolic, metabolically quiescent, high AMPK activity	Long-term survival, immune surveillance
Effector T Cell [81]	Aerobic Glycolysis	Anabolic, high glycolytic flux, high mTORC1 activity	Rapid proliferation, cytokine production, cytotoxicity
Memory T Cell [81,82]	OXPHOS, FAO	Enhanced mitochondrial biogenesis, high spare respiratory capacity	Long-term persistence, rapid recall response
Exhausted T Cell [63,74]	Dysfunctional OXPHOS and Glycolysis; ADO accumulation with suppressed A2A–mTOR signaling	Mitochondria fragmentation, reduced glycolytic and OXPHOS activity, bioenergetic failure	Diminished cytokine production, loss of proliferation capacity, sustained dysfunction

## 4. Strategic Metabolic Reprogramming of CAR T Cells

A growing body of evidence demonstrates that CAR structure profoundly influences T cell metabolic programming and function. The evolution of CAR T cell generations has been closely tied to advances in metabolic reprogramming and immune cell function. First-generation CARs, containing only the CD3ζ signaling domain, showed limited efficacy, in part due to poor metabolic support for proliferation and persistence. Second-generation CARs introduced costimulatory domains such as CD28 or 4-1BB, which enhanced glycolytic activity or mitochondrial respiration, respectively, resulting in increased T cell expansion and survival. Third-generation CARs included dual costimulatory domains, further boosting metabolic fitness and anti-tumor activity. Fourth-generation CARs (“TRUCKs”) integrated additional transgenes, such as cytokine-encoding sequences, augmenting metabolic responses and resistance to the suppressive tumor microenvironment. Notably, CARs incorporating CD28 costimulatory domains tend to promote glycolytic metabolism, driving rapid proliferation and effector differentiation, but may also be associated with reduced persistence [83]. In contrast, CARs utilizing 4-1BB signaling domains enhance oxidative phosphorylation and mitochondrial biogenesis, which supports long-term survival and memory formation [84,85]. Additionally, design features such as tonic signaling—constitutive activation of the CAR in absence of antigen—can result in metabolic exhaustion and impaired anti-tumor efficacy [83]. Other structural elements, including the affinity of the single-chain variable fragment (scFv), hinge and spacer regions, and transmembrane domain, modulate CAR expression, signal transduction, and ultimately the metabolic status and functionality of CAR T cells [86]. Understanding these relationships is critical for refining CAR design to optimize cellular metabolism, persistence, and anti-tumor activity.

**Figure 1:**
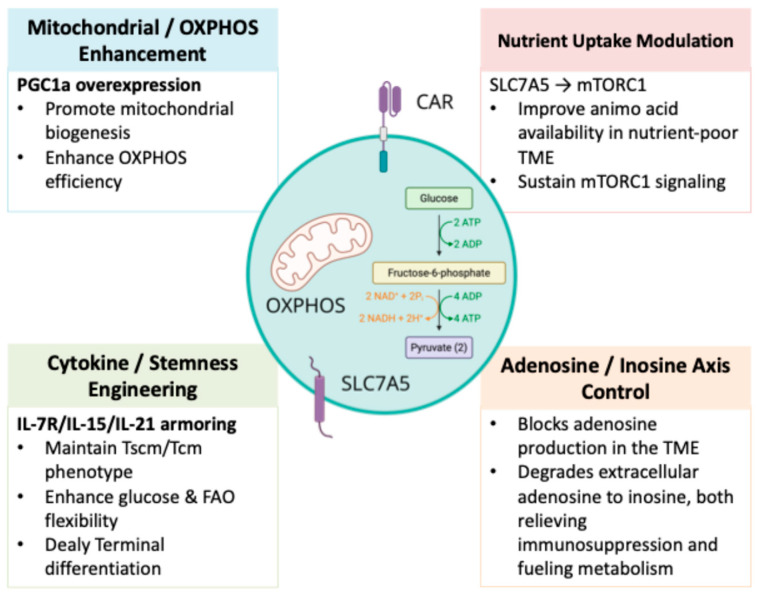
Integrated Metabolic Engineering Strategies to Enhance CAR T Cell Fitness in Solid Tumors. The figure summarizes four major categories of metabolic interventions that collectively aim to improve CAR T cell fitness within nutrient-restricted, immunosuppressive tumor microenvironments. Mitochondrial/OXPHOS enhancement, exemplified by peroxisome proliferator-activated receptor-gamma coactivator 1α (PGC1α) overexpression, promotes mitochondrial biogenesis and oxidative phosphorylation, strengthening persistence but potentially increasing tonic stress. Cytokine and stemness engineering, including IL-7R (C7R) armoring and ex vivo conditioning with IL-15 and IL-21, helps maintain Tscm/Tcm phenotypes, enhances metabolic flexibility, and delays terminal differentiation. Nutrient-uptake modulation, such as SLC7A5 overexpression, improves amino acid transport and sustains mTORC1 signaling in nutrient-poor environments, though it may not fully compensate for glucose depletion. Control of the adenosine/inosine axis, achieved through CD73 blockade, ADA1 expression or fusion strategies, A2A receptor knockout, or inosine supplementation, reduces adenosine-mediated suppression while providing inosine as an auxiliary carbon source to support glycolysis and OXPHOS. Together, these metabolic engineering strategies provide complementary avenues to enhance CAR T cell metabolic resilience, persistence, and antitumor activity in solid tumor settings. Arrows in the schematic denote directional biological relationships, including metabolic flux (such as glucose conversion to pyruvate and its entry into OXPHOS), signaling directionality (e.g., SLC7A5 driving mTORC1 activation), and functional enhancement (e.g., PGC1α promoting mitochondrial biogenesis). The ATP/ADP and NAD^+^/NADH labels indicate the points of energy production or cofactor reduction within glycolysis and oxidative phosphorylation. All arrows represent qualitative directionality rather than quantitative magnitude.

The formidable challenge posed by the metabolically hostile tumor TME has catalyzed the development of a sophisticated arsenal of strategies aimed at genetically and functionally reprogramming CAR T cells to resist metabolic suppression [87]. This endeavor moves beyond the traditional focus on antigen-recognition domains and signaling modules to engineer the very core biochemical processes that sustain T cell function. The goal is not merely to enhance a single pathway but to instill a state of metabolic plasticity—the capacity to dynamically adapt fuel utilization to prevailing conditions—thereby promoting durability, stemness, and sustained effector function. These interventions can be broadly categorized into intrinsic genetic rewiring, extrinsic pharmacological modulation, and combinatorial approaches that target the TME itself (Figure 1, Table 3).

### 4.1. Genetic Engineering for Intrinsic Metabolic Fitness

The most direct and stable approach involves genetically modifying CAR T cells to express factors that enhance metabolic performance or delete genes that impose metabolic constraints. A cornerstone strategy focuses on enhancing mitochondrial biogenesis and function. The master regulator of this process is PGC1α. Forced expression of PGC1α in CAR T cells drives a broad pro-mitochondrial program, resulting in increased mitochondrial mass, improved membrane potential, enhanced spare respiratory capacity, and greater reliance on oxidative phosphorylation. Preclinical models have demonstrated that PGC1α-overexpressing CAR T cells exhibit superior persistence, reduced expression of exhaustion markers (such as PD-1 and TIM-3), and significantly improved control of solid tumors, particularly in hypoxic and nutrient-poor conditions [74,88,89]. This metabolic rewiring effectively promotes a phenotype reminiscent of long-lived memory T cells. Complementing this approach is the modulation of exhaustion-associated transcription factors. The NR4A family (NR4A1, NR4A2, NR4A3) is upregulated in exhausted T cells and can repress mitochondrial function. Genetic knockout of NR4A genes using CRISPR-Cas9 technology has been shown to ameliorate mitochondrial dysfunction, enhance effector cytokine production, and confer resistance to exhaustion, leading to robust anti-tumor activity in vivo [90].

A second major genetic strategy involves rewiring nutrient sensing and utilization pathways to circumvent specific barriers in the TME. A critical target is the potent immunosuppressive ADO–A2AR axis. Pharmacological inhibition of A2aR renders CAR T cells impervious to the cAMP-mediated suppression triggered by extracellular ADO [91,92]. This strategy allows CAR T cells to maintain robust cytotoxicity and cytokine production even within adenosine-rich tumor niches, a common feature of many solid malignancies. A complementary approach is to endow CAR T cells with ADA1, either through surface tethering or secreted fusion formats [12,34,54,56,77,93]. ADA1 converts immunosuppressive ADO into inosine, a metabolically useful nucleoside that can feed central carbon metabolism through glycolysis and the TCA cycle. In this way, CAR T cells not only escape adenosine-mediated suppression but also gain access to a novel nutrient stream, directly transforming an immunosuppressive metabolite into an energy substrate.

To combat the pervasive problem of nutrient deprivation, CAR T cells can also be engineered to overexpress specific nutrient transporters. For example, overexpression of SLC7A5, which encodes a transporter for essential amino acids including leucine, ensures sufficient intracellular amino acid levels to sustain mTORC1 signaling—a critical driver of T cell activation and metabolism—even in an amino acid-poor environment [94,95]. This can prevent the anergy and functional impairment that otherwise results from nutrient scarcity.

Beyond directly targeting metabolic pathways, a powerful method to enhance overall metabolic fitness is through cytokine receptor armoring. Engineering CAR T cells to express a constitutively active chimeric cytokine receptor, such as the IL-7 receptor, provides a potent tonic pro-survival and pro-metabolic signal [96]. IL-7 signaling promotes glucose uptake and metabolism, enhances mitochondrial biogenesis and Bcl-2 expression, and critically, helps maintain cells in a less differentiated, stem-like state (Tscm). CAR T cells expressing C7R have demonstrated dramatic enhancements in persistence, metabolic capacity, and the ability to mediate complete regression of established solid tumors in mouse models, highlighting how leveraging native cytokine signaling pathways can broadly enhance metabolic fitness.

### 4.2. Pharmacologic and Ex Vivo Preconditioning Strategies

An alternative or complementary approach to permanent genetic modification is the transient metabolic manipulation of CAR T cells during the ex vivo manufacturing process using specific culture conditions or small molecules. A key advancement has been the shift in cytokine conditioning. Moving away from traditional expansion protocols reliant on IL-2, which promotes terminal effector differentiation and a metabolically inflexible glycolytic state, toward cultures using cytokines like IL-7, IL-15, and IL-21 helps generate CAR T products enriched for Tcm or Tscm phenotype [33,97]. These cytokines promote oxidative metabolism and mitochondrial biogenesis, resulting in cells with superior in vivo persistence and recall capacity.

Furthermore, the addition of specific metabolic modulators to the culture media can precondition cells for the TME. The AMPK activator metformin can be used ex vivo to enhance oxidative metabolism and favor memory formation [11,73]. Conversely, temporarily inhibiting glycolysis with 2-deoxy-D-glucose can force cells to rely on and expand their mitochondrial capacity, potentially enriching the final product for a metabolically resilient subset capable of better resisting the glucose-depleted TME. Importantly, inosine supplementation during ex vivo expansion has emerged as a simple but effective strategy to enhance T cell bioenergetics. Inosine can serve as an alternative carbon source to sustain ATP generation and nucleotide biosynthesis when glucose is scarce, and can synergize with ADA1-modified CAR T cells by ensuring continuous inosine availability.

### 4.3. Combinatorial Approaches: Remodeling the TME

Recognizing that engineering the T cell alone may be insufficient, the most clinically viable strategy will likely involve combining metabolically armored CAR T cells with agents that directly neutralize or remodel the immunosuppressive metabolic TME. This includes co-administering small molecule inhibitors that target key immunosuppressive pathways. For instance, CD73 inhibitors can prevent the generation of adenosine, thereby protecting both engineered and endogenous T cells [48]. Similarly, IDO1 inhibitors can block tryptophan catabolism and the production of kynurenines, while arginase inhibitors can prevent arginine depletion [37,38,39]. COX-2 inhibitors (e.g., celecoxib) can reduce levels of prostaglandin E2 (PGE2), another potent immunosuppressive metabolite [98].

A more integrative approach within this axis is to combine CD73 inhibition with ADA1-equipped CAR T cells or inosine supplementation, simultaneously suppressing adenosine production, degrading residual adenosine, and providing metabolic fuel. This three-pronged approach reframes adenosine from a purely suppressive metabolite into a usable nutrient, effectively flipping a metabolic liability of the TME into an advantage for CAR T cells.

A more indirect strategy involves the metabolic disruption of tumor cells themselves. Using drugs that target the unique metabolic dependencies of specific tumors (e.g., OXPHOS inhibitors, glutaminase inhibitors) could slow tumor growth and potentially reduce the tumor’s nutrient consumption, indirectly “freeing up” metabolites in the TME for use by CAR T cells.

The convergence of immunology and metabolism has thus unveiled a vast new toolkit for cellular engineering. The strategic integration of these approaches—arming CAR T cells with intrinsic metabolic advantages while simultaneously dismantling the extrinsic metabolic barriers erected by the tumor—represents the most promising path forward for achieving durable efficacy in solid tumors.

**Table 3:** Key Strategies for Metabolic Reprogramming of CAR T Cells.

Strategy Group	Molecular Target/Approach	Proposed Mechanism	Key Challenges
Mitochondrial/OXPHOS Enhancement [62,63]	PGC1α overexpression (PPARGC1A)	PGC1α overexpression (PPARGC1A)	PGC1α overexpression (PPARGC1A)
Cytokine/Stemness Engineering [33]	IL-7R (C7R) armoring	Promotes stemness, enhances glucose metabolism, prevents differentiation.	Risk of tonic signaling to exhaustion or transformation.
	Culture with IL-15/IL-21	Favors memory-like, oxidative metabolism phenotypes during manufacturing.	Standardization and scalability of ex vivo conditioning.
Nutrient Uptake Modulation [13]	SLC7A5 overexpression	Increases leucine import, sustaining mTORC1 activity in nutrient-poor TME.	May not address other nutrient limitations (e.g., glucose).
Adenosine/Inosine Axis Control [47,54,56]	CD73 inhibition (e.g., AB680)	Blocks adenosine production in the TME.	Combination therapy needed; possible off-tumor effects.
	ADA1 enzyme strategy (CAR T expression or fusion)	Degrades extracellular adenosine to inosine, both relieving immunosuppression and fueling metabolism.	Balancing enzymatic activity; immunogenicity concerns.
	A2A receptor knockout (ADORA2A KO)	Prevents T cells from sensing/suppressive signaling of adenosine.	Risk of unchecked T cell activation and off-tumor toxicity.
	Inosine supplementation	Supplies alternative carbon source (glycolysis + OXPHOS) under glucose restriction.	Optimization of dose; possible interference with nucleotide metabolism.

## 5. Challenges, Clinical Translation, and Future Perspectives

The compelling preclinical rationale for metabolically reprogramming CAR T cells is undeniable, and early proof-of-concept studies provide a robust foundation for optimism. However, the translation of these sophisticated bioengineering strategies from promising murine models to safe, effective, and reproducible human therapies is a formidable undertaking, replete with scientific, manufacturing, and regulatory hurdles. This section critically examines the multifaceted challenges inherent in this endeavor and outlines the future directions that will ultimately determine the clinical success of this paradigm.

### 5.1. Navigating Safety and Toxicity in Metabolically Enhanced Cells

Engineering cells for superior fitness and persistence fundamentally alters their biological behavior, inevitably raising complex safety considerations that must be prospectively and meticulously addressed. The primary concern is the potential exacerbation of on-target, off-tumor toxicity. Metabolically armored CAR T cells, equipped with enhanced cytotoxicity and resistance to exhaustion, could mediate more severe and prolonged damage if they recognize even low levels of target antigen on healthy tissues. For example, targeting ERBB2 (HER2) with a metabolically supercharged CAR T cell could potentiate catastrophic toxicity against HER2-expressing lung or heart tissue [99,100,101]. This risk necessitates the co-development of more sophisticated safety switches. While suicide genes like inducible caspase 9 (iCasp9) remain an option, the future lies in precision logic-gated systems. These include circuits that require the recognition of two antigens (AND-gate CARs) or the absence of a healthy tissue-specific antigen (NOT-gate CARs) for full activation, thereby adding a layer of protection that is absolutely critical for potentiated cells.

A more insidious theoretical risk is that of autonomous proliferation or autoimmunity. Disrupting intrinsic metabolic brakes—such as knocking out NR4A transcription factors or the A2AR—could lower the general threshold for T cell activation. While the intent is to overcome tumor-mediated suppression, there is a conceivable risk of aberrant, antigen-independent tonic signaling, leading to uncontrolled clonal expansion or the breaking of peripheral tolerance, triggering autoimmune phenomena. Extensive long-term toxicology studies in immunocompetent humanized mouse models or other advanced systems are non-negotiable to rule out these possibilities. Furthermore, enhancing T cell fitness and function could potentiate the magnitude and duration of cytokine production, potentially increasing the incidence or severity of cytokine release syndrome (CRS) and immune effector cell-associated neurotoxicity syndrome (ICANS). The clinical management protocols for these toxicities, including the timing and dosing of tocilizumab (IL-6R blockade) and corticosteroids, may need to be re-evaluated and preemptively adjusted for these more potent products.

### 5.2. The Daunting Hurdles of Manufacturing and Translation

The implementation of complex metabolic engineering poses profound practical challenges for cGMP-compliant production, potentially affecting cost, accessibility, and reproducibility. The foremost challenge is manufacturing complexity. Incorporating additional genetic modifications—for instance, overexpressing both ADA1 and a safety switch—adds multiple layers of complexity to the already intricate CAR T cell manufacturing process. Each genetic manipulation requires optimization to ensure high efficiency without compromising cell viability, genomic stability, or ultimate function. The use of multiple viral vectors or integrating CRISPR-Cas9 systems raises concerns about genotoxicity, oncogenic transformation, and unpredictable clonal dynamics. The reproducibility and scalability of these multi-step processes for large-scale, multi-center clinical trials remain unproven.

This complexity directly impacts the definition of Critical Quality Attributes (CQAs). For regulatory approval, a therapy’s CQAs—the biological markers that define its safety and efficacy—must be clearly defined. For metabolically reprogrammed CAR T cells, this extends far beyond standard metrics like transduction efficiency and CD4:CD8 ratios. It necessitates the development and validation of novel metabolic biomarkers as potential release criteria. These could include functional assays measuring mitochondrial spare respiratory capacity (Seahorse assays), basal glycolytic rates, or specific intracellular metabolite levels quantified by mass spectrometry. Validating these complex, often live-cell assays in a cGMP environment for batch release is a significant and unprecedented regulatory challenge.

### 5.3. Confronting Tumor Heterogeneity and Advancing Personalization

The metabolic profile of the TME is not monolithic; it exhibits significant inter- and intra-tumor heterogeneity. A one-size-fits-all metabolic armoring strategy may therefore be insufficient. The metabolic barriers in a highly hypoxic glioblastoma are distinct from those in a fibrotic, adenosine-rich pancreatic ductal adenocarcinoma or a lactate-acidotic melanoma. This heterogeneity mandates a move toward personalized metabolic profiling. Future clinical trials could involve stratifying patients based on metabolic features of their tumors, analyzed via deep biopsy. Immunohistochemistry for HIF-1α, CD73, IDO1, and other markers, coupled with metabolomic analysis of tumor interstitial fluid, could identify the dominant immunosuppressive mechanism(s) in a given patient. This would enable a rational, biomarker-driven approach to selecting a pre-designed CAR T product—for instance, administering ADA1-armed cells to a patient with a CD73-high tumor or PGC1α-overexpressing cells to a patient with a profoundly hypoxic tumor.

### 5.4. Future Directions: Toward Intelligent and Dynamic Systems

The future of this field lies in moving beyond constitutive, “always-on” metabolic engineering toward smarter, more dynamic, and integrated systems that respond to the environment. The next frontier is the integration of synthetic biology to create metabolic biosensors. Imagine a “smart” CAR T cell engineered with a hypoxia-response element (HRE) driving the expression of a payload gene, such as a adenosine inhibitor or a VEGF trap. Only upon infiltrating the hypoxic TME would the cell produce the drug, locally remodeling its immediate surroundings to create a positive feedback loop for its own survival and function. This concept of localized, condition-dependent production of therapeutic agents represents a monumental leap forward.

Furthermore, systems biology and multi-omics approaches will be crucial. Recent advances in sequencing technologies and bioinformatic analysis have greatly enhanced our understanding of CAR T cell biology and metabolism. For example, single-cell RNA sequencing has revealed substantial heterogeneity within CAR T cell populations, identifying distinct metabolic and functional states that can influence therapeutic efficacy and persistence [102,103]. Bulk transcriptomic analyses have linked gene expression profiles related to metabolic fitness with clinical outcomes in patients receiving CAR T therapy [104]. Furthermore, bioinformatic platforms such as TIMER3 facilitate the systematic evaluation of immune cell infiltration and metabolic gene signatures within the tumor microenvironment, offering insights into the interactions between CAR T cells and their targets [105,106]. These approaches collectively provide a more nuanced and quantitative framework for understanding the metabolic requirements and challenges faced by CAR T cells, and their integration with clinical and functional data promises to guide future optimization strategies. Integrating single-cell RNA sequencing, ATAC-seq, proteomic, and metabolomic data from patient tumors and CAR T products will identify novel metabolic targets and predictive biomarkers of response and toxicity. Machine learning algorithms can then integrate these massive datasets to help design optimal, patient-specific multi-gene engineering strategies. The ultimate goal remains the reliable and stable generation of Tscm cells, with their superior self-renewal capacity, longevity, and metabolic plasticity, representing the ideal therapeutic foundation. Future strategies will therefore focus on combining metabolic engineering (e.g., through PGC1α or IL-7R signaling) with targeted epigenetic modulation to lock CAR T cells into this most therapeutically desirable phenotypic state.

Despite its promise, CAR T cell therapy faces significant metabolic and clinical challenges. Off-target metabolic effects, such as unintended activation of metabolic pathways in non-target tissues, may be mitigated by the development of more tumor-selective CAR designs and the use of inducible or regulatable CAR systems. Tumor heterogeneity, which enables variable metabolic profiles and immune evasion, could be addressed by multi-targeted CARs or combination therapies that reshape the tumor microenvironment to be more permissive for CAR T cell activity. Manufacturing scalability remains a barrier; advances in automated cell culture, improved vector production, and allogeneic “off-the-shelf” CAR T products are promising developments. Finally, patient selection biomarkers—including metabolic profiling and gene expression signatures—may help identify individuals most likely to benefit from therapy, enabling personalized treatment approaches. These solutions warrant further investigation to improve both the efficacy and safety of CAR T cell therapy.

## 6. Conclusions

In conclusion, while the path is fraught with challenges, the strategic imperative is clear. Mastering CAR T cell metabolism is not a supplementary enhancement but a fundamental requirement for success in solid tumors. By confronting the translational hurdles with rigorous science, innovative engineering, and carefully designed clinical trials, the goal of creating truly effective “all-terrain” CAR T cells is within reach, promising to finally extend the revolutionary benefits of cellular immunotherapy to the vast majority of cancer patients.

## Data Availability

No new data were created or analyzed in this study. Data sharing is not applicable to this article.
